# One Host-Multiple Applications: Zebrafish (*Danio rerio*) as Promising Model for Studying Human Cancers and Pathogenic Diseases

**DOI:** 10.3390/ijms231810255

**Published:** 2022-09-06

**Authors:** Karolina Dudziak, Michał Nowak, Magdalena Sozoniuk

**Affiliations:** 1 Chair and Department of Biochemistry and Molecular Biology, Medical University of Lublin, 20-059 Lublin, Poland; 2 Institute of Plant Genetics, Breeding and Biotechnology, University of Life Sciences in Lublin, 20-950 Lublin, Poland

**Keywords:** zebrafish, xenograft, PDX, SCID, human disease

## Abstract

In recent years, zebrafish (ZF) has been increasingly applied as a model in human disease studies, with a particular focus on cancer. A number of advantages make it an attractive alternative for mice widely used so far. Due to the many advantages of zebrafish, modifications can be based on different mechanisms and the induction of human disease can take different forms depending on the research goal. Genetic manipulation, tumor transplantation, or injection of the pathogen are only a few examples of using ZF as a model. Most of the studies are conducted in order to understand the disease mechanism, monitor disease progression, test new or alternative therapies, and select the best treatment. The transplantation of cancer cells derived from patients enables the development of personalized medicine. To better mimic a patient’s body environment, immune-deficient models (SCID) have been developed. A lower immune response is mostly generated by genetic manipulation but also by irradiation or dexamethasone treatment. For many studies, using SCID provides a better chance to avoid cancer cell rejection. In this review, we describe the main directions of using ZF in research, explain why and how zebrafish can be used as a model, what kind of limitations will be met and how to overcome them. We collected recent achievements in this field, indicating promising perspectives for the future.

## 1. Introduction

In the past, the predominant model for the investigation of human diseases was based on 2D monolayer in vitro cell culture. Later, more studies used 3D cultures, spheroids, or organoids to mimic the human environment better. However, too many differences in drug responses between in vitro and in vivo models have indicated the huge need to develop in vivo models. Human tumors also interact with the surrounding tumor microenvironment (TME) and for this reason using animal models is the most reliable way to predict the individual patient response in a clinically meaningful manner. Nowadays, a combination of different model types, starting from monolayers, 3D cultures, in silico experiments and complemented with in vivo models, seem to give the fullest view of the studied issue.

In 2002, Amatruda et al. published a short paper showing a number of expected advantages of using *Danio rerio* (zebrafish, ZF) as a model, mostly for cancer studies [1].

Despite ZF and humans being almost on the opposite tops of the evolutionary tree, and their many anatomical differences, a number of facts show that zebrafish can be used as a model organism to study human health. Zebrafish shares extensive genomic homology with humans, and >80% of human genes associated with diseases are present in the zebrafish genome [2]. Moreover, the structure and the function of the majority of cancer-associated human genes are conserved between humans and zebrafish. Similar observations are found concerning the signaling pathways that control cell proliferation, migration, death, and differentiation. The system of mammalian vascular development regulation involving vascular growth factors and the respective receptors, as well as the signaling pathways, is also well conserved in the zebrafish model [3]. The best proof showing the significant role of this model in the research is the increasing number of publications that recently can be observed [4,5,6,7]. 

In comparison to mice, *Danio rerio* has many advantages that make it more useful in different types of research. Firstly, it is transparent, which enables the monitoring of labeled single cells or even the whole tumor. For scientists, doctors, and of course patients, time is very important. Zebrafish offers a model for detection in just 4 days, to observe the differences in behaviors at a single-cell resolution, differential proliferation rates, and metastatic and angiogenic potentials. Moreover, female zebrafish lay around 300 eggs/week. A short life cycle and a small size allow the study to be conducted much faster and on a bigger scale. Additionally, the similar response observed to some treatments in ZF and mice or patients suggests that ZF can be used instead of mice in some types of research and the results can be used for clinical therapy. Most studies use ZF in early life stage. However, the response to some treatments can vary compared to adults [8]. Embryos and larval stages do not fully show adult disease states because of their lack of a complete immune system [9]. For this reason, adults play a special role and promote translational research as a more reliable in vivo model.

**Figure 1:**
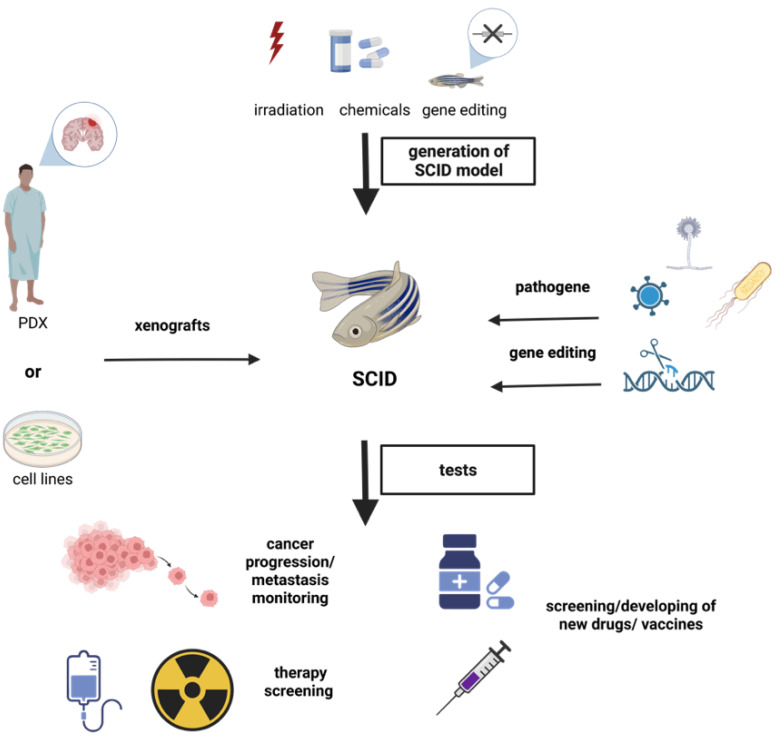
Avenues of research using ZF as a model to study human disease. This is an original figure created in BioRender.com (accessed on 28 July 2022).

In this review, we summarize the latest studies with the ZF model, which prove it is a very promising tool to study human diseases. The most common directions of studies using ZF as a model are presented in Figure 1.

## 2. Disease Induction: How to Create a Fish Model

### 2.1. Human Cell Transplantation

The general definition of xenografts states that it is the transplantation of an organ, tissue, or cells to an individual of another species [10]. Animals can play the role of the host for human cells derived directly from patients (PDX—patient-derived xenografts) or human cell lines. In zebrafish, the transplantation is easier to perform without rejection because of the absence of an immune system in the early stages; the fully mature system develops in 3–4 weeks [11].

#### 2.1.1. Cancer Cell Lines—Xenografts

The first experiment using a zebrafish model was performed with transplanted human cell lines. This is understandable because cell lines are more available, ‘clean’, well-characterized, their behavior is better known, and using them does not require so many ethical permissions. Moreover, tumors derived from patients have higher heterogeneity than laboratory-stable tumor cell lines. The first human cell line that was not rejected by the ZF recipient was the metastatic melanoma cell line, C8161 [12]. The injection was performed in ZF embryos. Many of them developed normally despite the presence of human cells. The melanoma cells survived, divided, and migrated. It is interesting that malignant melanoma cells were more migratory and disseminated to a wider variety of embryonic locations compared with the other, non-cancerous human cells used as a control. The next study was performed on a 48 hpf (hours post fertilization) embryo injected with medullary thyroid carcinoma (MTC) [13]. Most of the time, the injection is targeted into the yolk as it offers a nutrient-rich environment for transplanted cells. Since this first success, ZF has become more preferentially adopted as a model and has been injected not only during the embryonic stage but also in later developmental stages. The melanoma ZMEL1-GFP cell line was transplanted into the larval (vasculature of larval casper recipient) and adult zebrafish (orthotopically transplanted into the ventral skin of the transparent casper strain). For both, the same pattern of metastatic spread was reported, which is analogous to stage IV disease [14]. KSHV (Kaposi’s sarcoma-associated herpesvirus)-infected PEL (primary effusion lymphoma) cells injected into the yolk sac of larvae indicated proliferation and the embryos tolerated xenotransplantation [15]. Non-small cell lung cancer (NSCLC) H1299 (p53-deficient) and A549 (p53-wild type) cell lines were xenografted into larval stage [16], as well as osteosarcoma (U2-OS) cells [17]. Another transplantation method used Matrigel as a cell matrix. Multiple myeloma cell lines were resuspended in Matrigel and transferred to the perivitelline space of the ZF embryo [18].

In most studies, the transplanted cells are labeled, which allows control of the injection efficiency, monitoring of the cell localization, growth, development, and division, or even the presence of metastasis [19]. The injection can be performed with a single cancer cell. Using this method, two cell populations of leukemia stem cells, aldehyde dehydrogenase-positive (ALDH+) and -negative (ALDH), were injected into ZF and cell proliferation was observed [20]. 

#### 2.1.2. Cancer Cells Derived from Patient—PDX

As described before, the transplantation of patient tumors is much more complicated. However, the need to create PDX for the development of personalized medicine is so great that many studies are conducted with this aim. Even during the first successfully performed transplantation of the human melanoma cell line into zebrafish, researchers also transferred human fibroblasts and melanocytes to control cancer cell migration [12].

Zebrafish PDX (zPDX) is mostly used for cancer studies. The main role is to keep the tumor in the condition that is the most similar to the patient’s environment, monitor its behavior, and screen for the best therapy. Cancer cells are collected from patients during surgery or biopsy from primary tumors or metastasis. Then, cells are suspended, grown in the proper medium, and injected into ZF. The first successful zPDX was performed on pancreatic cancer metastasis cells to study the role of miR-10a [19]. Patient tumor cells are mostly, but not always, suspended. Pancreatic cancer-stained tissue fragments of approximately one-fifth to one-half the size of the yolk were transferred into the yolk for the impact on metastasis tests [19]. Primary GBM (glioblastoma) cells were cultured using three cell culture methods: organoid culture, neurosphere culture, and adherent culture, and then injected into the ZF. A comparison of these three culture types showed that attached culture GBM cells yielded the highest success rate, of 58% (attached cells) versus 25% (neurosphere) and 9% (organoid) [21].

Similar to that described for cell lines, cells derived from patients can also be injected into different body compartments and during different fish developmental stages. Human neuroendocrine tumor cells were injected into the developing subintestinal vein (SIV) plexus and common cardinal vein (CCV) in zebrafish embryos. Pro-angiogenic and invasive behaviors were observed 48 h after injection [22]. Other zPDX were described for a blood cancer patient with leukemia cells [23], human umbilical cord blood and human leukemia bone marrow samples [24], non-small cell lung cancer [25], pediatric diffuse midline gliomas [26], rhabdoid tumors, which is a pediatric tumor with very poor prognosis [27], and pancreatic [28] or human colon cancer [29]. The model obtained through patient-derived cells transplantation into the same organ or part of the ZF body is called orthotropic and it has been performed for brain tumor [30] or glioblastoma injection into the ZF’s midbrain [31,32].

#### 2.1.3. Beyond Cancers

Despite most studies being focused on cancers, many researchers have tried to introduce other cell types into animal models. As mentioned before, melanocyte cell lines and human fibroblasts were successfully transferred [12]. However, there are more examples of non-cancer cells. Among the thousands of different types of human immune cells, macrophages play the most important role. In living ZF, they can be induced from human primary monocytes that can later differentiate into macrophages. These types of cells stay alive even at the lower temperature characteristic for zebrafish (28.5 °C). Human monocytes injected into the brain and the circulatory system can stay in ZF for up to 2 weeks [33]. Zebrafish was also successfully used as a model to study hematopoiesis and hematopoietic stem cells (HSCs) that are responsible for the production of mature blood cells in bone marrow [34]. Conducting such types of studies and HSC transplantation is of significant importance in the therapy of patients with a variety of hematological malignancies [35]. Induced pluripotent stem cells (iPS cells) are another type of human cells that can be transplanted into ZF. Isolated iPSC-derived neural progenitor cells from human fibroblasts can be grafted into the embryo or 3-day-old fish. They can differentiate into neurons and survive in the recipients for more than 2 weeks [36]. 

### 2.2. Genetic Manipulation

Lung or breast cancer can only be modeled in zebrafish xenografts. In contrast, the induction of evolutionally conserved solid tumors, such as liver cancer, pancreatic cancer, colon cancer, and melanoma, can be obtained by using genetic engineering tools for gene editing or genetic manipulation. The most important commonly used transgenic lines are presented in Table 1.

#### 2.2.1. Introduction of Human Genes

The system for introducing human genes is well known and in most studies is based on the *tol2* system. This uses a plasmid with genes encoding human proteins under ZF promoters. Injection should be performed during the single-cell stage. Using this method, the first humanized zebrafish expressing human hematopoietic-specific cytokines (GM-CSF, SCF, and SDF1α) was obtained [24]. ZF expressing human *CXCL12* under the zebrafish *sdf1a* promoter was used for testing human leukemia cells [47]. Similarly, the function of human cytochrome P450 proteins was tested by introducing a plasmid with human CYP3A4 [48].

A method based on a tissue-specific promoter and genes involved in the oncogenic pathway allows the activation of certain cell types and has been well described for modeling certain cancer types. The expression of human AKT1 under the NBT promoter induces brain tumor within neural cells. Using a transgenic ZF line with labeled immune cells enabled the observation of the immune system response, which showed an increase in macrophages and other immune cells of the central nervous system, such as macroglia [49]. Analogous induction of the human myeloperoxidase expression enables analysis of the function of ZF neutrophils. The Lyz promoter is used for the expression of a tested protein in ZF neutrophils [50]. For the liver cancer induction of the hepatocyte-specific promoter, *fabp10a* has been extensively used as the tissue-specific driver and *kras^V12^* oncogene, which is crucial in driving liver tumorigenesis [51]. For pancreatic tumor studies, two transgenic zebrafish models have been developed: *Tg(ptf1a:eGFP-KRAS^G12V^)* [52] and *Tg(ptf1a:Gal4-VP16; UAS:eGFP-KRAS^G12V^)* [53]. Chronic myeloid leukemia was induced in the *tol2* system by introducing human BCR/ABL1 oncogenes. In transgenic *Tg(hsp70:p210^BCR/ABL1^)*, the numbers of *lcp1^+^* pan-myeloid cells, *lyz^+^* neutrophils, SB^+^ neutrophils, and *mfap4^+^* macrophages were significantly higher compared with the WT controls [54]. For neuroblastoma generation, the expression of MYCN oncogene combined with the expression of ALK (anaplastic lymphoma kinase) was induced [55]. Humanized transgenic ZF with human myeloperoxidase (MPO) in neutrophils was created to investigate the roles of MPO during infection and inflammation [50]. 

Another interesting approach is the generation of the tumors based on epigenetic regulation. Transgenic ZF with activity of human NUP98–HOXA9 (NHA9), a fusion oncogene found in high-risk acute myeloid leukemia, showed a direct link with overexpression of DNA (cytosine-5)-methyltransferase 1 (dnmt1). The expression of NHA9 caused an increase in the number of hematopoietic stem cells [56]. 

Finally, a very complex, double-humanized model has been designed via synthetic cells that combined humanization at the genetic and cellular levels (transgenesis and xenograft). The transgenic zebrafish HCC (hepatocellular carcinoma) model with conditional expression of human MET and induced ectopic Wnt signaling in hepatocytes (genetic level) was microinjected by human peripheral blood mononuclear cells (PBMCs) with a customized synthetic Notch receptor (synNotch) cascade to express the Wnt inhibitor DKK1 and anti-human MET (cellular level) [57].

#### 2.2.2. Mutations

It is well known that genetic mutations caused by environmental or endogenous stimuli promote cancer development. The generation of such genome changes in the ZF model is sometimes crucial for proper disease analysis. A broad knowledge of gene mutations in specific cancer allows the creation of the environmental characteristics for its development. In most cases of angiogenesis or cancer development, mutated genes are those involved in the tp53 or Wnt signaling pathways [58]. For this reason, a highly potent mutagen, N-ethyl-N-nitrosourea, was used for the mutation of the *tp53* gene [59] or *rag1* gene [60]. 

Genetic engineering is a much more powerful tool for creating mutations. Nowadays, the CRISPR system is commonly used for gene editing and designing transgenic models [61]. It is based on cutting double DNA in a very specific place with endonuclease Cas9. Then, the mutation can be induced in two ways:

(1) Altering the transcription by removing some crucial genes or part of genes—the ‘knock-out’ or
(2) Introducing genes involved in disease in the place of double break—the ‘knock-in’.

For the highest efficiency of knock out, the microinjection of guide RNA and the Cas9 protein complex should be performed in a one-cell stage embryo. Then, mosaic larvae can be directly used in the study, which is called a transient knock out approach. A longer process leading to obtaining a stable homozygous line is called an isogenic stable knockout. F0 larvae are crossed and generate heterozygous F1 and then F2 homozygous mutant larvae. It takes around 6 months but enables hundreds of F2 larvae to obtained, which can be used in drug screening as they have a more reliable biological background [62]. Many zebrafish mutant models have been developed through CRISPR/Cas9 [63]. Successful models have been established for neurological [64,65], kidney [66], and muscle defects [67] and orofacial clefts [62,68]. 

Before CRISPR, the main technique used for gene knockout was TALEN. Based on this method, a knockout of *gas7* promoting neuroblastoma metastasis in transgenic fish overexpressing *MYCN* was obtained [69]. Recently, TALEN has been used for the generation of immune-deficient models by targeting interleukin-2 receptor gamma common (IL-2Rγc) [70] or DNA-dependent protein kinase (Prkdc) [39].

The creation of knock-in mutants by recombination between fish genome and exogenous gene is based on one of two strategies of DNA repair pathways: homology-directed repair (HDR)-mediated recombination of a single-stranded oligodeoxynucleotide donor template (ssODN) or non-homologous end joining (NHEJ). Using HDR, a single-strand DNA sequence encoding a myc epitope tag (EQKLISEEDL) was introduced into the gene of TDP-43 zebrafish ortholog, which, in humans, is involved in the neurodegenerative diseases amyotrophic lateral sclerosis (ALS) and frontotemporal dementia (FTD) [71]. Similarly, four different knock-in lines were generated by combining CRISPR/Cas9 with a short template oligonucleotide leading to mutations in zebrafish orthologous of human genes involved in cardiovascular disorder (*abcc9, kcnj8,* and *pln*) [72]. However, the integration efficiency of the insert delivered as a plasmid or single-strand DNA with HDR is relatively low [62]. More efficient is NHEJ, which, on the other hand, can generate some INDEL. This method can be used to insert reporters or drivers in the genome. 

Most of the genes introduced into the ZF genome are transferred in the plasmid. However, the last study has shown more efficient CRISPR-Cas9-mediated knock-in can be obtained by using long ssDNA as a donor and without toxic effects [73]. Using the lssDNA donor template revealed a higher knock-in efficiency rate (~90%) compared to plasmid (~15%).

Another very powerful tool next to CRISPR, still commonly used in many studies, is gene silencing induced by anti-sense DNA analogs (morpholino). The knockdown of specific ZF genes caused by morpholino can directly provide the tumor microenvironment. The injection of a splice-blocking morpholino directed to knock down the LG-domain of lama5 caused the removal of the peptide region recognized by GBM for attachment. This ZF model enabled the observation of the behavior of transplanted human glioblastoma cells in microenvironments with and without lama5-binding sites. The knockdown of lama5 indicated an effect on the fish phenotype, the truncation of the yolk sac tail extension, and tail fin dysmorphogenesis. Lama5 caused a decrease in GBM cells spread by 23% and an increasing number of microtumors by the formation of blood vessels [74]. Morpholino targeted on Rps19 caused a deficiency of ribosomal proteins (RPs) in ZF and led to the development of Diamond Blackfan Anemia (DBA) associated with anemia, congenital defects, and cancer [75]. The knockdown of myd88 was used in the study of *Burkholderia cenocepacia* inflammation, which causes devastating pulmonary infections in patients with cystic fibrosis and chronic granulomatous disease [76]. 

For higher gene editing efficiency mediated by oncogenes or CRISPR-Cas9, an electroporation-based approach, the Transgene Electroporation in Adult Zebrafish (TEAZ) can be used. This method applies electrical pulses to generate pores within the cell membrane. Extracellular biomolecules, such as DNA, are able to cross the membrane and enter the cell. Using this technique, multiple transgenes can be transferred into specific anatomical locations in adult ZF. Successfully applied TEAZ was performed to generate malignant melanoma driven by expression of oncogenic BRAFV600E combined with the loss of the tumor suppressors tp53 and rb1 in ZF. The fish were injected with DNA constructs into the dorsal fin, and then the electrical pulses were applied by paddle-shaped electrodes across the injected region. This method can achieve a 100% success rate for transgene expression in the injected animals with 100% survival when performed in the dorsal skin [77]. 

Even though mutations caused by Cas9 or TALEN in the zebrafish models somehow mimic the aberrance of cancers, they are not the same as in humans. Most mutations of oncogenic patients are found exclusively in the focal tissues and not in the whole organism or organ [58]. From this point of view, the fish xenografts seem to be more reliable.

### 2.3. Pathogen Infection

The main purpose of introducing pathogens into ZF is to promote the immune response, generate immune cells, and study host–pathogen interactions. The immune system of *Danio rerio* is very similar to that of humans. Consequently, both the innate immune responses in larvae (1–2 weeks post fertilization), and adaptive responses in adults (after about 4–6 weeks) can be studied. Larvae or adult infection can be achieved by immersion, injection, or foodborne routes, depending on localization of the studied pathogen as well as the type of analyzed tissue [78]. In ZF, bacteria, fungi, and viruses have been extensively studied. 

The induction of bacterial infection can be performed by injection into the corresponding body compartments (tail fin, trunk) and at different developmental stages (embryo, larvae, adult). Zebrafish is a well-established model for studies of tuberculosis caused by *Mycobacterium tuberculosis* (*Mtb*) as it is naturally infected by *Mycobacterium marinum* (*Mm*). The genomes of *Mtb* and *Mm* share 3000 orthologs, with an average amino acid identity of 85% [79]. Studies based on the ZF model infected with *Mycobacterium* had a significant contribution to the changed view on the role of the granuloma in tuberculosis pathogenesis [80]. Infection of *Mn* can be achieved both in early-stage ZF (24–30 h post fertilization) by injection into the caudal vein or hindbrain ventricle [80] or adult fish by intraperitoneal injection [81]. Adults can also be infected by bath immersion or oral incubation [82]. Injection with *Mm* bacteria can also be done into the yolk, hindbrain, otic, notochord, subcutaneous, tail fin, or trunk depending on the goal of the research [83]. Another bacteria, *Mycobacterium leprae* (the causative agent of leprosy in humans) was used to study granulomas. The optimal temperature of growing for these bacteria is approximately 30 °C, which makes ZF a good model for these types of tests [84]. Urinary tract infection was tested by the injection of uropathogenic *Escherichia coli* (UPEC) from patient urine samples into the tail fin [85]. Humanized ZF with human myeloperoxidase was used for *Staphylococcus aureus* infection [50]. The inoculation of larval zebrafish with patient-derived *Vibrio cholerae* was used to study how the human pathogen invades the intestine [86]. Nowadays, a new method based on bath immersion has been developed and tested with *Pseudomonas aeruginosa*. The 48 h old ZF with tail injured were placed in plates with bacterial suspension [87]. Many other bacteria like *Burkholderia cenocepacia, Salmonella typhimurium* and *Shigella flexneri* have already been studied in ZF [88]. 

Transgenic lines with labeled immune cells are also useful to study the response to fungi. *Candida auris* (a serious threat to hospital patients) and *Talaromyces marneffei* (an important opportunistic pathogen in HIV patients) were injected into the hindbrain of ZF in the larval stage [89]. Fungi causing lung infection, such as *Aspergillus fumigatus* [90] and *Candida albicans* [89], have been injected into the zebrafish hindbrain.

Because of its many advantages, ZF has been a very important tool during the SARS-CoV-2 pandemic. Many papers describe ZF as a model for investigation of the mechanisms or searching for the perfect treatment against COVID-19 disease. In most studies, infection was promoted by the injection of the protein that causes symptoms, a SARS-CoV-2 spike (S) protein, leading to inflammation and olfactory dysfunction. One such study showed that only a fragment of S protein, a receptor binding domain (RBD) from S1 subunit caused a neurotoxic effect in adult fish leading to olfactory pathology and loss of smell [91]. The recombinant SARS-CoV-2 spike protein was also investigated on humanized ZF with xenotransplanted human alveolar epithelial cells (A549) [92] or in the posterior lobe of the swim bladder and used in drug tests [93]. 

Naturally, SARS-CoV-2 is not the only virus that has been tested in ZF. Alphaviruses, such as chikungunya virus (CHIKV) and Sindbis virus (SINV), which cause acute illnesses in humans, are sometimes associated with neuropathies, mostly in infants and elderly patients, were also introduced into ZF. Their impact on the central nervous system was examined by the injection of 70-72 hpf larvae with viral SINV or CHIKV particles. Injections have been performed in the caudal vein, aorta, optic tectum, and retina [94]. 

## 3. Aim of the Studies—What Is the Point?

The way in which the study is conducted and how the fish model looks depends on the main aim of the research. The investigation can have many different directions. Most of the studies are focused on cancer analysis and the search for the perfect therapy. However, there are many other directions that we wish to discuss here.

### 3.1. Cancer Analysis

Investigation of the genomic profile of cancer cells has revealed their heterogeneity. The analysis of the proliferation rate and metastatic and angiogenic potential in a patient with colon tumors indicated a number of differences, not only between patients, but also between different clones from the same tumor, even when mixed into a polyclonal tumor. This means cancer heterogeneity is not only between cancers (intertumor), but also within each cancer (intratumor) [29]. Using Incucyte, a new system for in vivo real-time cancer development monitoring, the growth, invasion, and survival can be observed. Of eleven cultures, three patient-derived glioblastoma cell cultures showed a high degree of association between grafted tumor cells and host blood vessels, suggesting a perivascular invasion phenotype [95].

An important direction of cancer research is metastasis analysis, which causes around 90% of all cancer deaths. Mouse mammary epithelial cells (EpH4) with oncogenic Ras (EpRas) transplanted into the ZF and treated by TGF-β (to induce the EMT—epithelial mesenchymal transition) exhibited metastasis in blood islands, brain, caudal fin, caudal vein, gill arches, heart, intestine, liver, mandible, optic cup (eye), otic cup, pericardium, somites, and swim bladder. Similarly, the primary tumor cells were shown to be invasive to the neighboring tissue, entered circulation, migrated, and homed in at distant tissues and organs [96]. In NSLC, ZF xenograft-induced metastasis accurately predicts lymph node involvement in patients [25]. For melanoma research, a zebrafish-specific melanoma cell line (ZMEL1) was used from transgenic zebrafish in which the melanocyte-specific mitfa promoter drives the human BRAFV600E gene. Isolated, unpigmented ZMEL1-GFP cells injected into the vasculature of larval casper zebrafish bypassed the initial skin site and formed widespread macrometastases after 7 days. A similar effect was observed for orthotopic ZF after 14 h of transplantation into ventral skin. The primary tumor mass becomes deeply pigmented and small anterior metastases formed in the fish [14]. Sometimes, the time of metastasis spread is so short that prediction of the place where they can occur is crucial. Fish xenografts with primary gastrointestinal human tumors indicated metastasis formation after 24 h of transplantation. Different metastatic profiles of pancreatic tumor cells revealed no metastasis after the injection of cloche mutant embryos with no vasculature [96]. The location of cell transplantation also plays an important role in tumor spread. The injection of a prostate cancer cell line into the yolk did not show metastasis. However, the injection of the same cells into the circulation leads to seed in the caudal hematopoietic tissue of the zebrafish tail where they proliferate [97]. A similar effect was observed for colon cancer transplanted into PVS and the circulation. The authors distinguished these two types of invasion in the early (invasion of surrounding tissues and intravasation into blood vessels) and later (survival in circulation, extravasation, and colonization) stages of the metastatic cascade [23]. 

The aim of testing cancers is very often to discover the function of genes. In this case, the commonly used method is (again) CRISPR, which enables editing of the genome and alters gene transcription [98]. For investigation of gene function, the most common methods are based on immunochemistry [99] or genes expression analysis by qPCR [98]. In cancer cells, such as any other, every event is promoted by a number of genes involved in many different signaling pathways. One of the most important systems is the MAPK cascade, which is activated by BRAF signals to promote the genesis of melanoma in fish with the BRAF^V600E^ mutation [99]. Moreover, the genetic interaction between the BRAF and p53 pathways leads to melanoma development. Similar dependency of these two pathways was observed in ZF with non-small cell lung carcinoma [16] and osteosarcoma [17]. CFTR (cystic fibrosis transmembrane conductance regulator) is involved in Wnt-dependent hematopoiesis, which interacts with adherens junction molecule AF-6/afadin via PDZBD (PDZ binding domain), thus regulating epithelial polarity and affecting cancer metastasis [100]. SF3B1 was overexpressed in human PDAC (pancreatic ductal adenocarcinoma) and associated with tumor grade and lymph-node involvement [101]. The inhibitor of nuclear factor kappa B kinase subunit epsilon (IKKε) is involved in the regulation of the stem cell phenotype in breast cancer cells [102]. Not only do enzymes play an important role in cancer signaling, but also other molecules such as non-coding RNA. The expression of long non-coding RNA (lncRNA) H19 and miR-675 tested in ZF xenografts with human breast cancer showed an increase in the invasive capacities [103]. The knowledge of a gene’s function in pathological cases can be very useful for the development of new therapies. 

### 3.2. Host–Pathogen Interactions

Because ZF has many advantages as an immune model, numerous papers described this animal in pathogen–host interaction. Moreover, there are many commonly available transgenic lines with labeled immune cells (macrophages or neutrophiles) that make these types of analyses even easier. 

A new role for macrophages has been shown using the ZF model for *Burkholderia cenocepacian* infection. These usually protective cells are critical for bacterial multiplication. In contrast, the neutrophil defense did not significantly contribute to disease outcome. *B. cenocepacian* can escape the protection provided by neutrophils. Moreover, infection with *B. cenocepacia* causes a fatal, pro-inflammatory response that partially depends on Il1 signaling [76]. *Bdellovibrio bacteriovorus,* which is a predatory bacteria from a range of Gram-negative bacterial pathogens, caused an increase in zebrafish survival during *Shigella* *flexner* infection. It has been suggested that *Bdellovibrio* works together with the host immune system, promoting pathogen killing, and can be used as a “living antibiotic” [104]. 

Using ZF, it was proved that bacteria spread from one granuloma to another by the departure of infected macrophages. This leads to the infection expansion and migration, both hematogenous and through tissues in early tuberculosis [80]. 

Tests of both *C. auris* and *C. albicans* in ZF showed a different immune response. After *C. albicans* infection, neutrophils started to produce NETs (neutrophil extracellular traps), which are structures of DNA, histones, and proteins with antimicrobial activity. In contrast, *C. auris* infection did not lead to NETs formation [89]. Gene expression analysis of the two kinases involved in the WNK signaling pathway (SPAK and OXSR1) revealed their upregulation during *Mycobacterium marinum* infection [98]. The mutation of *OXSR1* decreased bacterial burden and intracellular potassium levels. In OXSR1 activity, the NLRP3 inflammasome activation, caspase-mediated release of IL-1β, and downstream activation of protective TNF-α are involved. Other recent interesting achievements are presented in Table 2. 

### 3.3. Screening of Treatment Potential 

During the SARS-CoV-2 pandemic, ZF emerged as a very powerful rapid tool in drug tests and drug development. The viral spike protein has been described as part of the virus that causes inflammatory damage. Divya–Swasari–Vati medicine had a protective effect in A549 xenotransplanted zebrafish injected with the recombinant spike protein of SARS-CoV-2 [92]. However, more studies have been performed on ZF to screen therapies [27,62]. 

#### 3.3.1. Anticancer

Different responses to the most common chemotherapy against colon cancer based on FOLFIR and FOLFOX treatment were observed between primary and metastatic tumors. FOLIFIR led to significant induction of apoptosis and reduction of tumor mass only on lymph node metastases. Similar differences indicated heterogeneous colon tumor populations with different *KRAS* mutational status. HCT116 *KRAS* mutated cells are also more sensitive with FOLIFIR, as well as FOLFOX. Surprisingly, cetuximab, a drug that is not used in patients with *KRAS* mutation, induced apoptosis of HCT116 ZF xenografts only when it was tested alone; no effect was observed in combination with FOLFIR or FLOFOX [23]. ZF xenografts with the AGS and SGC7901 cell lines indicated that the highest potential against GC (gastric cancer) among well-known drugs (ramucirumab, apatinib, regorafenib, and cabozantinib) was cabozantinib, which had the lowest ED_50_ (effective dose, is the dose that produces a specified effect in 50% of the population under study) and highest anti-angiogenic and anti-proliferation activities and tolerated toxicity [112]. 

T cell immunotherapy using chimeric antigen receptor T (CAR T) cells, bispecific T cell engagers (BiTEs), and antibody peptide epitope conjugates (APECs) was conducted against leukemia. A direct correlation between T cell and tumor cell contact and killing was proved. Among them, the CAR T cells engaged with tumor cells as the early response. The interaction between CAR T cells and tumor cells elevated overall tumor cell death, causing a reduction in tumor cell number and a higher percentage of apoptotic synapse formation between a CAR T cell and tumor [113]. A screening of the whole drug set against rhabdoid tumor, embryonal RMS, and neuroblastoma revealed the best effect on the rhabdoid tumor of idasanutlin, ponatinib (RTK inhibitor), and tazemetostat (EZH inhibitor), as they caused a decrease in tumor progression. Against neuroblastoma, only one drug, ceritinib (an ALK inhibitor), had satisfactory effect, causing a decrease of 30% in the tumor size. Embryonal RMS responded to idasanutlin and navitoclax (BCL2 family inhibitor). Interestingly, no enhanced effect was observed with these two drugs together [27]. Widely used anti-CML (chronic myeloid leukemia) drugs—the tyrosine kinase inhibitors: imatinib, dasatinib, and bosutinib—effectively reduced the expanded myeloid population in humanized leukemic ZF *Tg(hsp70:p210^BCR/ABL1^*), which means this ZF model responds in the same way as humans. The screening of 171 new target drugs on this transgenic model found that 10 inhibitors (icaritin, CC-223, BEZ235, AZD3759, icotinib, DB07268, NQDI-1, selonsertib (GS-4997), LY364947, and ciliobrevin A (HPI-4)) inhibit *lcp1^+^* progression and potentially can be used in CML treatment [54]. Humanized ZF with CYP3A4 protein can be used for toxicity or metabolism analysis of the drug [48]. Marizomib, which is a proteasome inhibitor commonly used in clinical trials against glioblastoma, caused increased survival in transplanted ZF. The results indicated that the model will be useful for patient-specific drug testing and the best model would be based on 3013MG cell culture as a robust choice of a fast-growing PDC [95]. In medullary thyroid carcinoma, the anti-angiogenic effect of cabozantinib compared to vandetanib has been proved [13]. An interesting approach is testing epigenetic therapy. NHA9 fish embryos treated with a combination of DNMT or COX inhibitors with histone deacetylase (HDAC) inhibitors showed normal blood development in preleukemia state [56].

Another direction is using ZF as a tool for testing a new combination of well-known drugs to obtain a better anticancer effect. Ramucirumab, which is a human monoclonal antibody, and apatinib, a tyrosine kinase inhibitor, share the same therapeutic target, VEGFR-2, and they are used in gastric cancer therapy. Both agents indicate a better effect in combination with other drugs. Combining apatinib with ramucirumab together with docetaxel or 5-fluorouracil (5-FU) indicated an enhanced inhibitory effect on tumor cells metastasis and decreased tumor cell proliferation. The same effect of this drug combination was observed on zebrafish xenotransplanted with AGS and SGC-7901 cell lines and 70 zPDX. The inhibition of the tail metastasis of SGC-7901 cells was observed after treatment of all drugs together or separately [103]. In vivo analysis of the drug toxicity and neurotoxicity of ONC206 and ONC201 in pediatric diffuse midline gliomas (DMGs) proved that the combination of these two drugs was the most effective in prolonging survival [26]. A new, interesting direction in cancer research is testing the anticancer potential of newly discovered plant compounds. A theabrownin derived from green tea treatment caused the suppression of human non-small cell lung carcinoma when tested in a zebrafish xenograft [16]. Another interesting plant is *Radix Salviae miltiorrhizae* (also called Danshen in traditional Chinese medicine) containing tanshinol. The in vivo data show that tanshinol significantly suppresses osteosarcoma by apoptosis of U2-OS cells through a p53-mediated pathway [17]. Icaritin, which is a natural flavonoid derived from the traditional Chinese medicine epimedium, reduced the expanded myeloid population in chronic myeloid leukemia [54].

#### 3.3.2. Vaccination

Adult and larvae zebrafish can form granulomas that are very similar to those found in humans. Moreover, both the innate and adaptive immune responses generated during mycobacterial infection are composed of the same primary components. ZF, which naturally can be infected by *M. marinum,* is widely used as a model for the screening of vaccine candidates or for developing new vaccines against *M. tuberculosis*. Data obtained from zebrafish studies indicate that the classical BCG vaccination (the only available TB vaccine) can have the same protective effect against *M. marinum* infection as a newly developed DNA vaccinations. Four DNA vaccines based on *M*. *marinum* antigens with homologs in *M*. *tuberculosis* (CDP-diacylglycerol pyrophosphatase cdh, and two antigens belonging to the PE/PPE family and RpfE) showed a protective effect by reducing both, the mortality and bacterial counts, in a manner dependent on the adaptive immune response and enhanced production of IFN-γ [114]. Protection against a *M. marinum* infection in zebrafish was also observed using DNA-based vaccines with selected mycobacterial antigens: Ag85B, CFP-10, and ESAT-6 [115]. 

#### 3.3.3. Irradiation

Animal models are mostly used in drug tests of therapies against cancer. An alternative to chemotherapy is radiotherapy, which is also very effective in the treatment of some cancer types. ZPDX was already used in such tests with colorectal cancer. Analyses of mitosis, apoptosis, tumor and nuclear area size showed that HCT116*^KRASG13D^* cells are sensitive to radiation, whereas their isogenic cells, Hke3*^KRASwt^* are resistant [116]. However, irradiation can not only be used in anticancer therapies. A low dose of IR promotes the acceleration of sprouting angiogenesis, which can induce tumor growth and metastasis [117]. Further, γ-irradiated wild-type adult zebrafish with transplanted melanoma cells displayed extensive invasion of metastatic melanoma at the site of injection and the invasion of multiple organs, including the gut, heart, liver, pancreas, and kidney marrow, and possible vascular invasion [99].

### 3.4. Personalized Medicine

Despite progress in targeted cancer treatments, there is still no method to predict a specific cancer response to the therapy applied. To select the best treatment, patients must go through rounds of trial-and-error approaches that very often cause unnecessary side effects. The most perspective direction seems to be using ZF for creating patient-derived xenografts. Testing new drugs, drug combinations, or other alternative therapy on animal models and then applying the best treatment for the individual patient would be a perfect solution for personalized medicine. For the first time in 2015, ZF was used as a host for patient cells for T-cell acute lymphoblastic leukemia. This study presented a screening of drugs targeting NOTCH and the PI3K/AKT/mTOR signaling axis. No correlation between fish and patients has been described [23]. However, the latest studies show promising results comparing the fish and patient responses, proving ZF to be a good tool in pre-clinical studies. ZF xenografts with human triple-negative breast cancer treated with bevacizumab showed a correlation with clinical resistance/progression. In two cases, no significant impact was observed on the induction of apoptosis in the tumor [3]. A very successful study shows NSLC tumor regression in zPDX after paclitaxel and erlotinib treatment. The results corresponded to mouse PDX models and patients themselves. This suggests that ZF can be used instead of the mice model and ZF can be established directly from patient samples and accurately predict patient treatment outcomes [25]. Colorectal cancer tumor derived from patient biopsies treated with a single dose of 25 Gy (grays) in ZF showed induction of apoptosis. Most importantly, the results of ZF response correlated with the response of matching patients with rectal cancer to neoadjuvant chemoradiotherapy. This means that zebrafish avatars may indeed predict the clinical response to neoadjuvant therapy [116]. zPDX can also be useful in the prediction of cancer relapse. Among five zPDX with colon cancer treated with FOLFOX, four are anticipated to relapse or to have no relapse within 3 months to 6 months after surgery [29]. 

All these studies proved that zPDX is a promising in vivo model to select the best treatment in a personalized manner.

## 4. Limitations—How to Overcome Them?

Undoubtedly zebrafish is an attractive alternative and has huge potential to replace mice in preclinical studies in drug/therapies tests, and to be applied in personalized medicine. However, there are still many issues in which rodents show their predominance, mostly due to their higher conserved mammalian genome and biological processes and for this reason the fish and mice could be used in parallel [118]. Among the various advantages of ZF, there are limitations that researchers are intensively working on developing new methods to overcome most of them. 

The biggest problem with using an animal as a model for disease studies is their immune system, which works normally, produces immune cells, defends against the extreme conditions, and does not mimic the exact patient response. In the first study using ZF with human melanoma cell line as a model, it was noticed that the zebrafish microenvironment led to suppression of the tumorigenic phenotype of malignant melanoma cells [12]. For this reason, SCID (severe combined immunodeficient) models were developed. Few methods have been described to suppress the immune response. As the first, irradiation was used to prevent xenograft rejection [119]. After irradiation of transgenic lck:EGFP ZF, no T cells were observed. However, a single dose of irradiation caused an increase in lethality rates. Moreover, after a few weeks, the ZF immune system can recover, and the tumor can be rejected. A more effective method is based on chemical suppression using dexamethasone (DEX). Xenografts of human breast cancer cell line (MGSO-3) treated with DEX showed a 50% reduction in lymphocytes. This environment does not change tumor growth, morphology, viability, or even cell number [120]. This chemical suppressor has been successfully tested in xenotransplanted ZF with fluorescent human breast (MDA-435), sarcoma (HT1080) [121], and brain tumor [30] cells. A more sophisticated method is gene editing based on the mutation of crucial genes involved in immune cell formation. The first mutant SCID ZF has been developed by a team of Dr. David Langenau from Massachusetts General Hospital in Boston. They induced mutations of three genes: recombination activating gene 2 (*rag2*), dnA-dependent protein kinase (*prkdc*), and janus kinase 3 (*jak3*). Only the *prkdc* mutant reproduced as homozygotes and survived after transplantation of normal and tumor ZF tissues.

The results of histology, RNA sequencing, and single-cell transcriptional profiling showed a decrease in mature T and B cells in *prkdc*-deficient ZF [122]. This model has been successfully used to engraft human leukemia cells and drug tests [123]. Another model based on *prkdc*−/−, *il2rga*−/− zebrafish has been used as PDX for melanoma, breast cancer embryonal rhabdomyosarcoma (ERMS), and glioblastoma. Preclinical RMS analysis showed sensitivity to a combination of olaparib and temozolomide. Interestingly, these results have been confirmed in the SCID mice model [40]. 

However, animals with stable immune deficiency are very sensitive to some infections and they need to stay in a super clean and sterile environment. For ZF, such a completely clean environment is hard or even totally impossible to obtain. One option is supplementation with an antibiotic which, again, has an impact on the response of transplanted human cells or the treatment. For these reasons, there is a need to develop conditional SCID models with controlled immune deficiency (e.g., by temperature or chemicals). Another problem concerning the immune system is a lack of endogenous human T, B, NK cells, and macrophages, which precludes the real assessment of their impact on tissue microenvironment and response to therapy. To overcome this problem, a humanized ZF with the expression of multiple human hematopoietic-specific cytokines (CXCL12/SDF1α and the human SCF/KITLG and GM-CSF/CSF2-) has been developed. In the presence of these crucial cytokines, human transplanted leukemia cells exhibit hematopoietic niche homing, which is a better model to mimic the real human leukemia behavior. Moreover, such a model indicated the prolonged survival and differentiation of human HSPC [24]. 

In the past, one of the most common problems with ZF was the optimal temperature, which for *Danio rerio* is 28.5 °C. Nowadays, many studies have shown that ZF is more tolerant and can live at a higher temperature during different development stages. At 2 dpf, fish xenografts with patient NLSC cells incubated at 36 °C did not present any toxic phenotypes or reduced survival (compared to incubation at 28.5 °C). This temperature profile provides the best growth conditions for the transplanted tumors [25]. Maintenance of fish at 34 °C for the whole experimental period (from the embryo stage for 4 days) has also been successfully applied [3]. For in vivo microscopy, larvae were maintained at 33 °C to provide optimal conditions for bacterial ST144-YFP replication strain [85]. The last study showed that after acclimatization (32 °C on 1 to 2 d, 35 °C on 3d, 35.5 °C on 4 d, 36 °C on 5 d, 36.5 °C on 6 d, and 37 °C on 7 d) prkdc−/−, il2rga−/− fish can be maintained at 37 °C even for 6 months [40]. This enables a long-term single-cell visualization of cancer cell engraftment.

Another problem is creating ZF mutants. To increase the efficiency of HDR-mediated knock-in of ssDNA, an alternative for Cas9 is Cpf1 DNA nuclease derived from *Lachnospiraceae bacterium* ND2006. This improved approach for targeted DNA integration with the zebrafish genome can be four times more efficient than the Cas9 [124]. Another option is to try lssDNA, as it indicated a higher efficiency rate of integration with fish genome [73]. 

As we mentioned before, the response for treatment can vary between early life stage and adult fish. However, using adults is linked with many limitations (the irritation of exposed mucosal surfaces such as the eyes or gills, not applied for water-insoluble compound, unknown final drug concentrations). Other methods, such as oral gavage or injection, are invasive and require repeated anesthesia. All these techniques can be applied for short-term tests, but they can cause stress in the longer term. A recent study showed an interesting alternative based on supplementation with the drug in the food pellets. Addition of vemurafenib and application to ZF with BRAFV600E melanoma resulted in the reduction of the tumor growth. This drug pellet method enables the control of the drug dose and long-term application in adult fish [9].

Some authors described that the small size of the ZF embryo does not allow recovery of the tumors after a period of growth and that it is impossible to investigate the next stages of tumor progression and metastatic development beyond 3 days or the high mortality after injection [25]. However, we should keep in mind that studies performed on ZF can be performed on hundreds of ZF embryos in a single experiment. Despite genetic approaches often being sufficient, the limited availability of zebrafish-specific antibodies causes difficulties in using immunofluorescence or Western blotting techniques [78]. 

In summary, it is easy to observe that ZF is perhaps not a perfect model and there is still much to work on. However, the results of extended studies conducted in the direction of its development offer a very promising perspective.

## Figures and Tables

**Table 1:** Selected zebrafish transgenic lines commonly used in human disease studies.

Transgenic Line	Characteristics	Use	References
Tg(mpeg1.1:Dendra2)	labeled macrophages	monitoring immune response	[37]
Tg(lyz:EGFP)	labeled neutrophils	monitoring immune response, investigation how lysozyme-expressing cells participate during inflammation	[38]
Prkdc −/−	decrease of immune cells formation	as a SCID model	[39]
Prkdc −/− il2rga −/−	decrease of immune cells formation	as a SCID model	[40]
*nf1a^†/−^*; *nf1b^†/−^*; *ptena^†/−^*; *ptenb^†/−^*; *p53^†/M214K^*	loss of NF1/PTEN	for melanoma analysis	[41]
Lck:GFP	cell-specific tyrosine kinase (*lck*) promoter labeled T cells	tracking of T cell (development and ablation)	[42]
Tg (*fli-1*: EGFP)	expressing enhanced green fluorescent protein (EGFP) in endothelial cells	GFP-labeled vasculature	[43]
roy −/−, nacre −/−	Casper line—without pigmentation	This line, which was named *casper* for its ghost-like appearance, demonstrates a complete lack of all melanocytes and iridophores in both embryogenesis and adulthood.	[44]
*Tg(kdrl:mCherry)*	endothelial cells	vascular-specific reporter line	[45]
*Tg(gata1a:GFP)*	red blood cells labeled	thrombocytes activity	[46]

**Table 2:** Examples of pathogen infections tested in zebrafish models.

Pathogen	Findings	References
*Pseudomonas aeruginosa*	Developed a new method for *P. aeruginosa* infection in zebrafish embryos based on bath immersion, thus avoiding the time-consuming microinjection step.	[87]
*Pseudomonas aeruginosa*	OprF protects *P. aeruginosa* against macrophage clearance during acute infection, by avoiding destruction in phagolysosomes.	[105]
*Mycobacterium marinum*	Zebrafish embryos carrying a mutation in *Myd88*, the common adaptor of Toll-like and interleukin-1/18 receptors, have increased susceptibility to *M. marinum* infection following intravenous injection.	[106]
*Mycobacterium marinum*	Rifampicin-loaded nanoparticles are significantly taken u [by macrophages and lead to improved embryo survival during tuberculosis].	[107]
*Mycobacterium marinum*	Mycobacteria inside infected macrophages secrete the ESAT6 virulence factor, involved in promoting nearby epithelial cells to secrete Mmp9 (matrix metalloproteinase), which can increase the migration of macrophages.	[108]
*Escherichia coli*	L-forms could provide a source of bacterial survivors during treatment with cell wall-specific antibiotics during urinary tract infection.	[85]
*Talaromycosis marneffei and Aspergillus fumigatus*	Conidia initially phagocytosed by neutrophils were transferred to macrophages. β-Glucan from *fungi* cell walls can promote fungal dissemination by host cell shuttling.	[109]
*Kaposi’s sarcoma associated-herpesvirus (KSHV)*	Development of a new platform for modeling KSHV-associated diseases. Model can be used as a PDX.	[15]
*Staphylococcus epidermidis*	IL-6 signaling protects zebrafish larvae during *S. epidermidis* infection. Macrophages play a primary role in the host immune response and are involved in the clearance of infection. A potential role of miR-142-5p–*il-6st* interaction in this infection model.	[110]
*Mycobacterium marinum*	OXSR1 inhibits inflammasome activation by limiting potassium efflux during mycobacterial infection.	[98]
*Escherichia coli*	Monopoiesis and granulopoiesis occurred following systemic bacterial infection.	[111]
